# Divergent warning patterns contribute to assortative mating between incipient *Heliconius* species

**DOI:** 10.1002/ece3.996

**Published:** 2014-02-23

**Authors:** Richard M Merrill, Audrey Chia, Nicola J Nadeau

**Affiliations:** 1 Department of Zoology, University of CambridgeCambridge, U.K; 2 Department of Animal and Plant Sciences, University of SheffieldSheffield, U.K

**Keywords:** Behavioral isolation, ecological speciation, magic traits, male preference, Nymphalidae

## Abstract

Theoretical models suggest that traits under divergent ecological selection, which also contribute to assortative mating, will facilitate speciation with gene flow. Evidence for these so-called “magic traits” now exists across a range of taxa. However, their importance during speciation will depend on the extent to which they contribute to reproductive isolation. Addressing this requires experiments to determine the exact cues involved as well as estimates of assortative mating in the wild. *Heliconius* butterflies are well known for their diversity of bright warning color patterns, and their amenability to experimental manipulation has provided an excellent opportunity to test their role in reproductive isolation. Here, we reveal that divergent color patterns contribute to mate recognition between the incipient species *Heliconius himera* and *H. erato*, a taxon pair for which assortative mating by color pattern has been demonstrated among wild individuals: First, we demonstrate that males are more likely to attempt to mate conspecific females; second, we show that males are more likely to approach pinned females that share their own warning pattern. These data are valuable as these taxa likely represent the early stages of speciation, but unusually also allow comparisons with rates of interbreeding between divergent ecologically relevant phenotypes measured in the wild.

## Introduction

Uncovering the mechanisms by which divergent mating behaviors evolve is a key to our understanding of animal diversity. In particular, theoretical models suggest that the speciation process is greatly facilitated if traits under divergent ecological selection also contribute to nonrandom mating (Gavrilets 2004). These so-called “magic” (Gavrilets 2004; Servedio et al. 2011) or “multiple-effect” traits (Smadja and Butlin 2011) evade the homogenizing effects of recombination, which impede the evolution of behavioral isolation when gene flow persists (Felsenstein 1981). Although the epithet “magic” was perhaps intended to suggest that these types of trait were rare in nature, accumulating evidence suggests that this might not be the case (e.g., Podos 2001; Puebla et al. 2007; Reynolds and Fitzpatrick 2007; Feulner et al. 2009; Conte and Schluter 2013). Nevertheless, the extent to which magic traits contribute to speciation remains unclear, and this will depend on the degree to which they contribute to reproductive isolation (i.e., their “effect size” sensu Nosil and Schluter 2011) (Servedio et al. 2011; see also Haller et al. 2012). As such, studies in which we can combine estimates of assortative mating in the wild with experiments to determine the cues involved will be especially valuable.

The neotropical butterfly genus *Heliconius* is well known for its diversity of bright warning color patterns, often associated with Müllerian mimicry (Müller 1879; Merrill and Jiggins 2009). The amenability of these color patterns to experimental manipulation has provided an excellent opportunity to test their role in reproductive isolation, and it has been argued that *Heliconius* provide the strongest empirical support for “magic traits” (Servedio et al. 2011). Specifically, the divergent patterns of *Heliconius cydno* and *H. melpomene* have been experimentally shown both to be under strong disruptive selection due to predation (Merrill et al. 2012) and to be used during mate recognition (Jiggins et al. 2001). Further behavioral studies across the continuum of divergent taxa in *Heliconius* have already contributed to our understanding of ecological speciation (Jiggins et al. 2001, 2004; Kronforst et al. 2006; Estrada and Jiggins 2008; Melo et al. 2008; Muñoz et al. 2010; Merrill et al. 2011a,b2011b). Nevertheless, there has been little opportunity to study the cues used during mate recognition between diverging taxa where assortative mating in the wild has also been considered. This makes it difficult to estimate the contribution of divergent wing patterns to premating isolation and their overall importance as magic traits for speciation.

The sister-taxa *Heliconius himera* and *H. erato* likely represent an intermediate step on the continuum from race to species. These incipient species share a narrow hybrid zone (∼5 km) in southern Ecuador, where *H. himera* replaces the more broadly distributed *H. erato* in the dry thorn-scrub habitats of the Andean valleys. In addition to warning color pattern, the taxa differ in development time, adult diurnal activity, and egg-laying rates, which may reflect adaptations to altitude-associated habitat shifts (McMillan et al. 1997; Davison et al. 1999). However, all hybrid and backcross offspring produced in the insectary are viable and fertile (McMillan et al. 1997). Furthermore, in areas of overlap, both species fly together and females oviposit on the same host plants (Jiggins et al. 1997b). Nevertheless, and in contrast to interracial contact zones within *H. erato,* parental types predominate in the hybrid zones between *H. erato* and *H. himera*, where, in addition to color pattern loci, mtDNA and allozyme loci also remain distinct (Jiggins et al. 1997a).

The lack of hybrid inviability implies ecological, and/or behavioral factors explain the deficit of hybrids in the contact zone between *H. himera* and *H. erato*. Frequency-dependent selection against rare hybrid or immigrant warning color patterns, as demonstrated in other *Heliconius* taxon pairs (Mallet and Barton 1989; Merrill et al. 2012), in addition to selection imposed by the abiotic environment, will likely contribute to the integrity of the species (Mallet et al. 1998b). In addition, both insectary experiments and estimates from wild individuals have revealed strong assortative mating between the species (McMillan et al. 1997; Mallet et al. 1998a). The evolution of these two barriers is likely connected. First, by increasing the rarity of hybrid forms, assortative mating would simultaneously increase the efficacy of mimetic selection as an isolating barrier [because predators are less likely to recognize rare forms as distasteful (Mallet and Barton 1989; Merrill et al. 2012)]. Second, if color patterns are also used during mate recognition, divergent ecological selection acting on this trait may strengthen assortative mating, via “by-product” (Schluter 2001) or “reinforcement-like” mechanisms (Servedio and Noor 2003; see also Jiggins et al. 2001; Kronforst et al. 2007).

These processes will be constrained by the breakdown of linkage disequilibrium between genes under divergent selection and those underlying assortative mating; however, this constraint disappears if the same trait underlies both processes (Gavrilets 2004). Although it has previously been shown that *H. himera* and *H. erato* mate assortatively both in the wild and the insectary (McMillan et al. 1997; Mallet et al. 1998a), it has not yet been demonstrated experimentally that warning patterns are used as mating cues. Here, we address this gap and present evidence that divergent color patterns contribute to assortative mating between these incipient species: First, we demonstrate that males are more likely to attempt to mate conspecific females; second, we show that males are more likely to approach pinned females that share their own warning pattern.

## Materials and Methods

*Heliconius himera* were collected from Vilcabamba and *H. erato cyrbia* from Balsas, in southern Ecuador. These sites are on either side of the narrow hybrid zone where the species meet (see Jiggins et al. 1996 for details of collecton sites and location of the contact zone). Males and females used in insectary trials were the offspring of stocks established from these wild caught individuals (<3 generations in captivity). We first tested whether *H. erato* and *H. himera* males were more likely to court (sustained hovering or chasing) and attempt to mate (where males bend their abdomens toward a female so as to copulate) conspecific rather than heterospecific females in 15-min no-choice trials. In each trial, a single mature male (>10 days after eclosion) was introduced into a cage (75 × 120 × 160 cm) containing a single virgin female (either *H. erato* or *H. himera* no more than 48 h since eclosion). We used generalized linear mixed models (GLMMs, implemented using the R package lme4), with binomial response and logit link function, to test whether males were more interested in conspecific females. Due to a limited number of individuals available, both males and females were used multiple times. Consequently, replicates were individual trials, with a bivariate response, and both male id and female id were included as random factors in our GLMMs to avoid pseudoreplication. Significance was determined by likelihood ratio tests (LRT) examining the change in deviance following the removal of a term describing whether the individual trial involved conspecific or heterospecific butterflies.

To test whether color pattern acts as a mate recognition cue, dead mounted *H. erato* and *H. himera* females were presented to males during 1-h trials. These trials were carried out in a 150 × 120 × 160-cm cage containing five to eight male *H. erato* and *H. himera,* each with a unique identification number on their forewing*. H. erato* from western Ecuador has an iridescent blue color that cannot easily be replicated artificially. This limited us to the use of real wings rather than artificial models. To remove cuticular hydrocarbons, pinned female butterflies were washed for 5 min in hexane – this was done for all trials with one exception. Females were presented attached to wires 80 cm apart and ∼1 m above the insectary floor. Female type and male id were recorded for all male approaches within 5 cm of the mounted females. Trials were repeated so that cumulative scores of the number of approaches toward each female type were obtained for each male included in the experiment. We estimated the relative probability of male approaches directed toward *H. himera* rather than *H. erato* for each male type using likelihood (Jiggins et al. 2001; Merrill et al. 2011b). The likelihood function was:


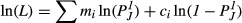


where *m*_*i*_* *= the total number of approaches by male *i* directed toward *H. himera, c*_*i*_* *= the total number of approaches by male *i* directed toward *H. erato,* 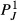 = probability of males of species *j* approaching *H. himera*. Probabilities of male approaches were estimated by numerically searching for values of 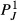 that maximized ln(*L*), using the solver option in EXCEL (Microsoft). To test whether *H. himera* and *H. erato* males responded differently to the mounted females, we first produced a model where relative probabilities for two species were set equal (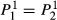). This was then compared to a second model in which relative probabilities for each genotype were estimated separately (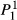 ≠ 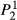) using a likelihood ratio test (LRT), where 2Δln(*L*) asymptotically follows a chi-square distribution with one degree of freedom.

## Results and Discussion

**Figure 1:**
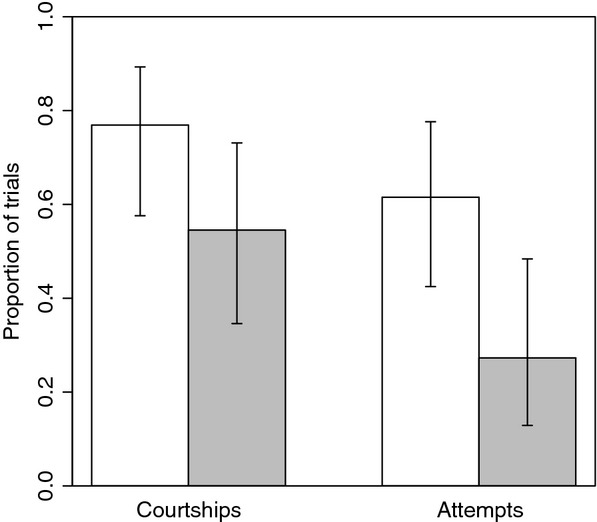
Proportion of no-choice trials involving conspecific (white bars) and heterospecific pairs (grey bars) in which males courted and attempted to mate females (+95% CIs)

Overall, our experimental results reveal differences in male behaviors that likely contribute to assortative mating detected between *Heliconius himera* and *H. erato* among wild individuals (Mallet et al. 1998a). In particular, we show that males differ in their responses toward con- and heterospecific females, and that divergent color patterns contribute to assortative mating between these taxa. In our no-choice trials with live butterflies (Table 1), males were more likely to attempt to mate conspecific females (LRT: 2Δln*L *= 5.28, df* *= 1, *P *=* *0.022; Fig. 1). The same trend was apparent, although less pronounced, in our analysis of courtship events, which necessarily precede attempted matings (Fig. 1). Although the trend was not significant in our GLMM analysis (LRT: 2Δln*L *= 2.62, df = 1, *P *=* *0.106), of 48 no-choice trials with live butterflies, courtship was observed between 77% of those involving conspecific individuals as opposed to 55% of the remaining trials involving heterospecific individuals (Appendix S1). Although assortative mating between *H. himera* and *H. erato* has been previously demonstrated through an extensive series of insectary experiments (McMillan et al. 1997), this study did not consider male and female behaviors seperately. As such, the results from our no-choice trials not only reinforce the previous conclusions reached by McMillan et al. (1997) but also demonstrate that male preferences contribute to assortative mating between these incipient species.

**Table 1:** No-choice trials with live butterflies in which courtship and mating attempts were observed.

	Male	Female	Number trials	Courtship	Mating attempt
Conspecific	*Heliconius himera*	*Heliconius himera*	7	4	3
	*Heliconius erato*	*Heliconius erato*	19	16	13
Heterospecific	*Heliconius himera*	*Heliconius erato*	10	5	3
	*Heliconius erato*	*Heliconius himera*	12	7	3

**Figure 2:**
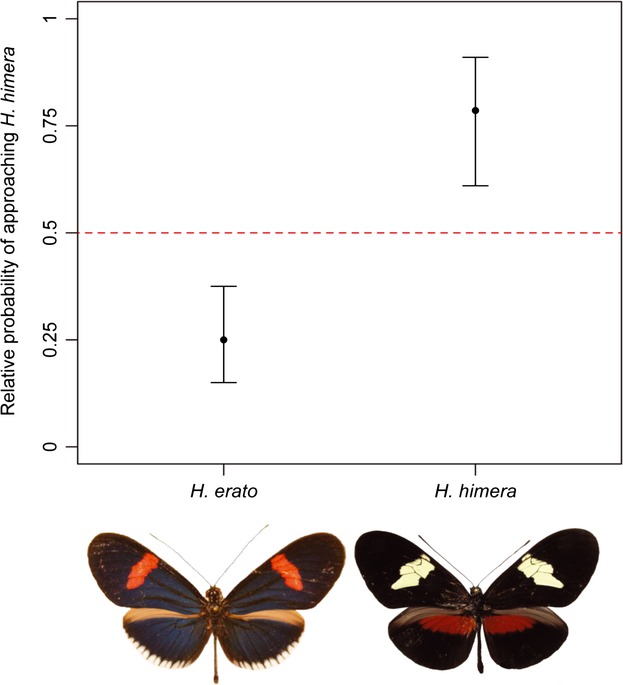
The relative probability of approaching *Heliconius himera* mounted females by *H. erato cyrbia* and *H. himera* males (below left and right, respectively), where one would indicate a complete preference for *H. himera* and 0 a preference for *H. erato cyrbia*. Dashed red line represents a relative probability of 0.5 (i.e., no preference). Support limits are asymptotically equivalent to 95% confidence intervals and were obtained by searching for values that decreased ln(L) by two units. Note that warning color patterns are sexually monomorphic.

Our experiments with mounted females reveal that *H. himera* and *H. erato* differ in their response to divergent warning patterns (Fig. 2; LRT: 2Δln*L *= 23.18, df = 1, *P *<* *0.001). Overall, *H. himera* males were less active and, compared to *H. erato,* were less likely to approach either mounted female (Appendix S2). Nevertheless, males of both species showed a preference and were more likely to approach mounted females that share their own color pattern (LRT comparing estimated probabilities to 0.5 (i.e., no preference): *H. himera,* 2Δln*L *= 9.72, df = 1, *P *<* *0.01; *H. erato,* 2Δln*L *= 15.70, df = 1, *P *<* *0.001). After exclusion of data collected in the single trial where mounted females were not washed in hexane, preference for conspecific wing patterns remained significant for both *H. himera* males (LRT: 2Δln*L *= 9.63, df = 1, *P *<* *0.01) and *H. erato* males (LRT: 2Δln*L *= 11.79, df = 1, *P *<* *0.001). Previous experiments with *Heliconius erato* have failed to detect a difference in approach rates toward wings washed with hexane and controls (Muñoz et al. 2010), suggesting that color pattern is the predominant cue.

Previous work on assortative mating in *H. himera* and *H. erato* did not explore the cues involved (McMillan et al. 1997). Indeed, McMillan et al. (1997) noted that convergence in color pattern between distant-related *Heliconius* species, due to selection for mimicry, would seem to make it a “poor signal for sexual communication” (see also Estrada and Jiggins 2008). Although our results suggest that this statement may have been premature, any conclusions clearly depend on our ability to control for confounding factors. Previous studies investigating the role of color pattern in *Heliconius* mating have used photographs, or real wings with manipulated color patterns (e.g., Jiggins et al. 2001; Kronforst et al. 2006). These may differ from unmanipulated real wings in aspects of hue and brightness, especially with respect to butterfly vision (Zaccardi et al. 2006; Bybee et al. 2012), and may even introduce further confounding factors by acting as “super stimuli”. Nevertheless, they can be used to confirm that butterflies are responding to color pattern rather than some other stimulus (Jiggins et al. 2001, 2001, 2004, 2004; Muñoz et al. 2010). Unfortunately, in the present study, we were restricted to the use of real wings alone. *H. erato* from western Ecuador has an iridescent blue color that cannot easily be replicated artificially, and experimental evidence suggests that such structural colors may be important in *Heliconius* mating decisions (Sweeney et al. 2003). In an attempt to remove chemical cues, mounted females were washed with hexane in all but one of our trials. Estrada and Jiggins (2008) report that *H. erato* males can distinguish between wings dissected from conspecific and heterospecific (although comimetic) *H. melpomene* females, but that this effect disappears after wings have been washed in hexane. In contrast, in experiments comparing mate preferences between races *H. erato*, Muñoz et al. (2010) found few differences between trials conducted with hexane-washed and nonwashed wings (one exception concerned the apparent ability of *H. erato chestertonii* to discriminate wings dissected from hybrids before but not after hexane treatment). Whether or not the lack of differences between hexane-washed and nonwashed wings reflects a failure of the experimental treatment or simply that there are few important differences between these closely related taxa remains unclear. However, considering the differences observed by Estrada and Jiggins (2008) the latter perhaps seems more likely.

Bearing in mind these potential limitations, our data contribute to a growing number of studies implying shifts in color pattern contribute to premating isolation between *Heliconius* taxa (Jiggins et al. 2001, 2004; Kronforst et al. 2006; Estrada and Jiggins 2008; Melo et al. 2008; Muñoz et al. 2010; Merrill et al. 2011b). However, our results are additionally valuable in that uniquely we can compare estimates of male preference for divergent color patterns to estimates of assortative mating for color pattern in the wild. The latter are not yet available for other *Heliconius* taxa pairs perhaps because hybrids are extremely rare (e.g., Jiggins et al. 2001), contact zones have been lost due to habitat destruction (e.g., Kronforst et al. 2006), or populations tested are not geographically adjacent (Estrada and Jiggins 2008). Estimating assortative mating in the wild is also easier in *H. erato*, where females tend to only mate once (Walters et al. 2012); in contrast, the majority of previous studies concern members of the *melpomene*-*cydno* clade (but see Estrada and Jiggins 2008; Muñoz et al. 2010), where multiple mating is more common.

By raising the offspring of wild females, in addition to wild eggs and larvae, sampled within the *himera-erato* hybrid zone, Mallet et al. (1998a) were able to infer the color pattern genotypes of the parents involved. Matings between heterospecifics were estimated at ∼5% (0.3–21.4%). In previous insectary experiments, which did not consider male (or female) preferences individually, matings between heterospecifics were 11% as common as between conspecifics. Using data from our experiments with mounted females, we can estimate that males approach heterospecific, relative to conspecific, color patterns at 24% (16–34%). The broad confidence limits permitted by our data, in addition to a number of potential caveats, including for example different light environments influencing “wild” and “insectary” mating decisions, command caution when interpreting these results. Nevertheless, there appears to be a deficit between our estimates and those from previous work, implying some influence of factors other than male preferences for color pattern on assortative mating. These may involve additional trait-preference interactions, including perhaps female preferences for color pattern, as well as pheromone and behavioral cues, all of which require further investigation in *H. himera* and *H. erato*, as well as *Heliconius* more generally.

One potentially important contribution to assortative mating may be interspecific differences in the cues and preferences associated with pupal mating. Although the frequency of this behavior in the wild remains unknown, *H. erato* has been observed to pupal mate; males patrol the forest searching for female pupae, so they can mate with uneclosed or freshly emerged females (Gilbert 1976). To date, this has only been explored as a source of reproductive isolation between *H. erato chestertonii* and *H. e. venus*, which are separated by a bimodal hybrid zone in the Cauca Valley, Colombia. In contrast to experiments with already eclosed females (where behavioral isolation was strong), in no-choice trails where males were presented with pupae, Muñoz et al. (2010) found no difference in the frequency of heterospecific and conspecific matings. However, in at least one other species of pupal-mating *Heliconius,* males are known to use both host plants and larvae to find potential partners (i.e., female pupae) (Estrada and Gilbert 2010). This interaction might influence the ability to distinguish between con- and heterospecific pupae. Indeed, Estrada and Gilbert (2010) demonstrate that *H. charitonia* males find plants on which *Agraulis vanilla* larvae are feeding less attractive than those with larvae of their own species. It would clearly be interesting to know whether males can distinguish between larvae of closely related *Heliconius,* such as *H. himera* and *H. e. cyrbia* or *H. e. chestertonii* and *H. e. venus*.

Recent genomic studies in *Heliconius* have revealed that gene flow between species is widespread (Heliconius Genome Consortium 2012; Martin et al. 2013; Nadeau et al. 2013). Traits that are under divergent selection and also act as mate recognition cues could play an important role in maintaining species differences in the face of this admixture. These types of traits are not unique to *Heliconius*, with an accumulating range of examples coming from other species (e.g., Podos 2001; Puebla et al. 2007; Reynolds and Fitzpatrick 2007; Feulner et al. 2009; Conte and Schluter 2013). The antagonism between selection and recombination may be overcome by a number of additional mechanisms, including, for example, pleiotropy (Maynard Smith 1966; as distinct from “magic traits” Smadja and Butlin 2011), close physical linkage (Felsenstein 1981), or learning (Servedio et al. 2009). Reproductive and ecological isolation may also require associations between additional traits (Hawthorne and Via 2001; Protas et al. 2008; Merrill et al. 2011b, 2013; Smadja and Butlin 2011). Nevertheless, accumulating data suggest that “magic traits” are widespread and may be important drivers of ecological speciation (Servedio et al. 2011; Nosil 2012), but to test this, we need to consider multiple points along the speciation continuum. This will allow us determine whether particular magic traits are early evolving components of premating isolation and consequently their importance for speciation. Our data for *H. erato* and *H. himera* are valuable in this regard as these taxa likely represent the early stages of speciation, but unusually also allow comparisons with rates of interbreeding between divergent ecologically relevant phenotypes measured in the wild.

## Supporting Information

Appendix S1 No-choice trials between pairs of *Heliconius himera and H. erato pairs*.

Appendix S2 Approaches toward *H. erato* and *H. himera* mounted females. ^*^Note that in trial 1, mounted females had not previously been washed in hexane. See main text for details.
