# Supplementary material for: Divergent warning patterns contribute to assortative mating between incipient Heliconius species
Source: Ecol Evol. 2014 Feb 23;4(7):911–7. doi: 10.1002/ece3.996 (PMC3997309; doi:10.1002/ece3.996)
Supplement: Appendix S1 — No-choice trials between pairs of Heliconius himera and H. erato pairs. [file ece30004-0911-sd1.docx]

**Appendix S1.** No choice trials between pairs of *Heliconius himera and* H. erato pairs.

| Male ID | Male Type | Female ID | Female Type | Courtship | Mating Attempt |
| --- | --- | --- | --- | --- | --- |
| E42 | *H. erato* | H1 | *H. himera* | Yes | Yes |
| E43 | *H. erato* | H1 | *H. himera* | No | No |
| E42 | *H. erato* | H1 | *H. himera* | No | No |
| E39 | *H. erato* | H2 | *H. himera* | Yes | Yes |
| E27 | *H. erato* | H2 | *H. himera* | No | No |
| E64 | *H. erato* | H2 | *H. himera* | Yes | Yes |
| E62 | *H. erato* | H2 | *H. himera* | No | No |
| E62 | *H. erato* | H3 | *H. himera* | Yes | Yes |
| E67 | *H. erato* | H7 | *H. himera* | Yes | Yes |
| E27 | *H. erato* | H7 | *H. himera* | Yes | Yes |
| E69 | *H. erato* | H7 | *H. himera* | Yes | Yes |
| E71 | *H. erato* | H7 | *H. himera* | No | No |
| H47 | *H. himera* | E1 | *H. erato* | No | No |
| H51 | *H. himera* | E1 | *H. erato* | No | No |
| H54 | *H. himera* | E1 | *H. erato* | No | No |
| H33 | *H. himera* | E2 | *H. erato* | Yes | Yes |
| H29 | *H. himera* | E3 | *H. erato* | Yes | Yes |
| H33 | *H. himera* | E3 | *H. erato* | Yes | Yes |
| H70 | *H. himera* | E6 | *H. erato* | Yes | Yes |
| H68 | *H. himera* | E6 | *H. erato* | No | No |
| H70 | *H. himera* | E8 | *H. erato* | No | No |
| H73 | *H. himera* | E8 | *H. erato* | Yes | Yes |
| E45 | *H. erato* | E1 | *H. erato* | Yes | Yes |
| E43 | *H. erato* | E1 | *H. erato* | Yes | Yes |
| E44 | *H. erato* | E1 | *H. erato* | No | No |
| E67 | *H. erato* | E2 | *H. erato* | Yes | Yes |
| E39 | *H. erato* | E2 | *H. erato* | Yes | Yes |
| E27 | *H. erato* | E2 | *H. erato* | No | No |
| E62 | *H. erato* | E2 | *H. erato* | Yes | Yes |
| E27 | *H. erato* | E3 | *H. erato* | Yes | Yes |
| E39 | *H. erato* | E3 | *H. erato* | Yes | Yes |
| E67 | *H. erato* | E3 | *H. erato* | Yes | Yes |
| E62 | *H. erato* | E3 | *H. erato* | Yes | Yes |
| E27 | *H. erato* | E6 | *H. erato* | Yes | Yes |
| E67 | *H. erato* | E6 | *H. erato* | Yes | Yes |
| E71 | *H. erato* | E6 | *H. erato* | Yes | Yes |
| E69 | *H. erato* | E6 | *H. erato* | Yes | Yes |
| E27 | *H. erato* | E8 | *H. erato* | Yes | Yes |
| E67 | *H. erato* | E8 | *H. erato* | Yes | Yes |
| E71 | *H. erato* | E8 | *H. erato* | No | No |
| E69 | *H. erato* | E8 | *H. erato* | Yes | Yes |
| H54 | *H. himera* | H1 | *H. himera* | Yes | Yes |
| H41 | *H. himera* | H2 | *H. himera* | Yes | Yes |
| H33 | *H. himera* | H2 | *H. himera* | Yes | Yes |
| H38 | *H. himera* | H2 | *H. himera* | No | No |
| H29 | *H. himera* | H2 | *H. himera* | No | No |
| H29 | *H. himera* | H3 | *H. himera* | Yes | Yes |
| H70 | *H. himera* | H7 | *H. himera* | No | No |
