# Supplementary material for: Divergent warning patterns contribute to assortative mating between incipient Heliconius species
Source: Ecol Evol. 2014 Feb 23;4(7):911–7. doi: 10.1002/ece3.996 (PMC3997309; doi:10.1002/ece3.996)
Supplement: Appendix S2 — Approaches toward H. erato and H. himera mounted females. *Note that in trial 1, mounted females had not previously been washed in hexane. See main text for details. [file ece30004-0911-sd2.pdf]

Appendix S2. Approaches towards *H. erato* and *H. himera* mounted females. *Note that in trial 1 mounted females had not previously ben washed in hexane. See main text for details.

	Male ID	Male Type	Trial Number	1*	2	3	4	5	6	7	8	9	10	11	12	13	14	15	16	17	18	19	20	21	22	23	24	25	26	27	28	29	30	31	33	34	35	Total
**Approaches towards *H. erato* model**	E27	*H. erato*		-	-	-	-	-	-	-	-	-	-	-	-	1	-	-	1	-	-	1	-	-	-	-	-	-	-	-	1	2	-	3	2	1	-	**12**
	E39	*H. erato*		-	-	-	-	-	-	-	-	1	-	-	-	1	-	-	-	-	2	1	2	1	-	-	1	1	1	-	-	-	-	-	-	-	-	**11**
	E42	*H. erato*		-	-	-	-	-	-	-	-	-	1	-	-	-	-	-	-	-	-	-	-	-	-	-	-	-	-	-	-	-	-	-	-	-	-	**1**
	E43	*H. erato*		2	-	-	-	-	-	-	-	-	-	-	-	-	-	-	-	-	-	-	-	-	-	-	-	-	-	-	-	-	-	-	-	-	-	**2**
	E45	*H. erato*		2	-	-	-	-	-	-	-	-	-	-	-	-	-	-	-	-	-	-	-	-	-	-	-	-	-	-	-	-	-	-	-	-	-	**2**
	E53	*H. erato*		1	-	-	-	-	-	-	-	-	-	-	-	-	-	-	-	-	-	-	-	-	-	-	-	-	-	-	-	-	-	-	-	-	-	**1**
	E62	*H. erato*		-	-	-	-	-	-	-	-	-	-	-	-	-	-	-	-	-	-	-	-	-	-	1	1	-	2	-	-	-	-	-	-	-	-	**4**
	E63	*H. erato*		-	-	-	-	-	-	-	-	-	-	-	-	-	-	1	-	-	-	-	-	-	-	-	-	-	-	-	-	-	-	-	-	-	-	**1**
	E67	*H. erato*		-	-	-	-	-	-	-	-	-	-	-	-	-	-	-	-	-	-	-	-	2	1	1	-	-	2	-	1	-	1	-	-	-	1	**9**
	E69	*H. erato*		-	-	-	-	-	-	-	-	-	-	-	-	-	-	-	-	-	-	-	-	-	-	-	-	-	-	-	-	-	-	-	1	-	1	**2**
	E71	*H. erato*		-	-	-	-	-	-	-	-	-	-	-	-	-	-	-	-	-	-	-	-	-	-	-	-	-	-	-	-	-	-	-	-	-	-	**0**
	H12	*H. himera*		-	-	-	-	-	-	-	-	-	-	-	-	-	-	-	-	-	-	-	-	-	-	-	-	-	-	-	-	-	-	-	-	-	-	**0**
	H26	*H. himera*		-	-	-	-	-	-	-	-	-	-	-	-	-	-	-	-	-	-	-	-	-	-	-	-	-	-	-	-	-	-	-	-	-	-	**0**
	H29	*H. himera*		-	-	-	-	-	-	-	-	-	-	-	-	-	1	-	-	-	-	-	-	-	-	-	-	-	-	-	-	-	-	-	-	-	-	**1**
	H33	*H. himera*		-	-	-	-	-	-	-	-	-	-	-	-	-	-	-	-	-	-	-	-	-	-	-	-	-	-	-	-	-	-	-	-	-	-	**0**
	H35	*H. himera*		-	-	-	-	-	-	-	-	-	-	-	-	-	1	-	-	-	-	-	-	-	-	-	-	-	-	-	-	-	-	-	-	-	-	**1**
	H38	*H. himera*		-	-	-	-	-	-	-	-	-	-	-	-	-	1	-	-	-	-	-	-	-	-	-	-	-	-	-	-	-	-	-	-	-	-	**1**
	H47	*H. himera*		-	-	-	-	-	-	-	-	-	-	-	-	-	-	-	-	-	-	-	-	-	-	-	-	-	-	-	-	-	-	-	-	-	-	**0**
	H51	*H. himera*		1	-	-	-	-	-	-	-	-	-	-	-	-	-	-	-	-	-	-	-	-	-	-	-	-	-	-	-	-	-	-	-	-	-	**1**
	H52	*H. himera*		-	-	-	-	1	1	-	-	-	-	-	-	-	-	-	-	-	-	-	-	-	-	-	-	-	-	-	-	-	-	-	-	-	-	**2**
	H56	*H. himera*		-	-	-	-	-	-	-	-	-	-	-	-	-	-	-	-	-	-	-	-	-	-	-	-	-	-	-	-	-	-	-	-	-	-	**0**
	H70	*H. himera*		-	-	-	-	-	-	-	-	-	-	-	-	-	-	-	-	-	-	-	-	-	-	-	-	-	-	-	-	-	-	-	-	-	-	**0**
	H54	*H. himera*		-	-	-	-	-	-	-	-	-	-	-	-	-	-	-	-	-	-	-	-	-	-	-	-	-	-	-	-	-	-	-	-	-	-	**0**
	H7	*H. himera*		-	-	-	-	-	-	-	-	-	-	-	-	-	-	-	-	-	-	-	-	-	-	-	-	-	-	-	-	-	-	-	-	-	-	**0**
	H39	*H. himera*		-	-	-	-	-	-	-	-	-	-	-	-	-	-	-	-	-	-	-	-	-	-	-	-	-	-	-	-	-	-	-	-	-	-	**0**
**Approaches towards *H. himera* model**	E27	*H. erato*		-	-	-	-	-	-	-	-	-	-	-	-	-	-	-	-	-	-	-	-	-	1	-	-	-	-	-	-	-	-	-	-	1	-	**2**
	E39	*H. erato*		-	-	-	-	-	-	-	-	-	-	-	-	-	-	-	-	-	1	-	-	-	-	-	2	-	1	1	-	-	-	-	-	-	-	**5**
	E42	*H. erato*		-	-	-	-	-	-	-	-	-	-	-	-	-	-	-	-	-	-	-	-	-	-	-	-	-	-	-	-	-	-	-	-	-	-	**0**
	E43	*H. erato*		-	-	1	1	-	-	-	-	-	-	-	-	-	-	-	-	-	-	-	-	-	-	-	-	-	-	-	-	-	-	-	-	-	-	**2**
	E45	*H. erato*		-	-	-	-	-	-	-	-	-	-	-	-	-	-	-	-	-	-	-	-	-	-	-	-	-	-	-	-	-	-	-	-	-	-	**0**
	E53	*H. erato*		-	-	-	-	-	-	-	-	-	1	-	-	-	-	-	-	-	-	-	-	-	-	-	-	-	-	-	-	-	-	-	-	-	-	**1**
	E62	*H. erato*		-	-	-	-	-	-	-	-	-	-	-	-	-	-	-	-	-	-	-	-	-	-	-	-	-	-	-	-	-	-	-	-	-	-	**0**
	E63	*H. erato*		-	-	-	-	-	-	-	-	-	-	-	-	-	-	2	-	2	-	-	-	-	-	-	-	-	-	-	-	-	-	-	-	-	-	**4**
	E67	*H. erato*		-	-	-	-	-	-	-	-	-	-	-	-	-	-	-	-	-	-	-	-	-	-	-	-	-	-	-	-	-	-	-	-	-	-	**0**
	E69	*H. erato*		-	-	-	-	-	-	-	-	-	-	-	-	-	-	-	-	-	-	-	-	-	-	-	-	-	-	-	-	-	-	-	-	-	-	**0**
	E71	*H. erato*		-	-	-	-	-	-	-	-	-	-	-	-	-	-	-	-	-	-	-	-	-	-	-	-	-	-	-	-	1	-	-	-	-	-	**1**
	H12	*H. himera*		-	-	-	-	-	-	-	-	-	-	1	-	-	-	-	-	-	-	-	-	-	-	-	-	-	-	-	-	-	-	-	-	-	-	**1**
	H26	*H. himera*		-	-	-	-	-	-	-	-	-	-	-	-	-	1	-	-	-	-	-	-	-	-	-	-	-	-	-	-	-	-	-	-	-	-	**1**
	H29	*H. himera*		-	-	-	-	-	-	-	-	1	-	-	-	-	-	-	-	-	-	-	-	-	-	-	-	-	-	-	-	-	-	-	-	-	-	**1**
	H33	*H. himera*		-	-	-	-	-	-	-	-	-	-	-	-	-	-	-	-	-	-	1	-	-	-	-	1	1	-	-	-	-	-	-	-	-	-	**3**
	H35	*H. himera*		-	-	-	-	-	-	-	-	-	-	-	-	-	-	-	-	-	-	-	-	-	-	-	-	-	-	-	-	-	-	-	-	-	-	**0**
	H38	*H. himera*		-	-	-	-	-	-	-	-	-	-	1	-	2	1	-	-	-	-	-	-	-	-	-	-	-	-	-	-	-	-	-	-	-	-	**4**
	H47	*H. himera*		2	-	1	-	-	-	-	-	-	-	-	-	-	-	-	-	-	-	-	-	-	-	-	-	-	-	-	-	-	-	-	-	-	-	**3**
	H51	*H. himera*		-	-	-	-	-	-	-	-	-	-	-	-	-	-	-	-	-	1	-	-	-	-	-	-	-	-	-	-	-	-	-	-	-	-	**1**
	H52	*H. himera*		-	-	-	-	1	-	-	-	-	-	-	-	-	-	-	-	-	-	-	-	-	-	-	-	-	-	-	-	-	-	-	-	-	-	**1**
	H56	*H. himera*		-	-	-	-	-	-	-	-	-	-	-	-	1	-	-	-	-	-	-	-	-	-	-	-	-	-	-	-	-	-	-	-	-	-	**1**
	H70	*H. himera*		-	-	-	-	-	-	-	-	-	-	-	-	-	-	-	-	-	-	-	-	-	-	-	-	-	-	-	-	1	-	2	-	-	-	**3**
	H54	*H. himera*		-	-	-	-	-	-	-	-	-	1	-	-	-	-	-	-	-	-	-	-	-	-	-	-	-	-	-	-	-	-	-	-	-	-	**1**
	H7	*H. himera*		-	-	-	-	-	-	-	-	-	-	-	-	1	-	-	-	-	-	-	-	-	-	-	-	-	-	-	-	-	-	-	-	-	-	**1**
	H39	*H. himera*		-	-	-	-	-	-	-	-	-	-	-	-	-	-	-	-	-	-	1	-	-	-	-	-	-	-	-	-	-	-	-	-	-	-	**1**
